# Psychometric assessment of the PROMIS Fatigue Short Form 6a in women with moderate-to-severe endometriosis-associated pain

**DOI:** 10.1186/s41687-020-00257-y

**Published:** 2020-10-27

**Authors:** Robin Pokrzywinski, Ahmed M. Soliman, Eric Surrey, Michael C. Snabes, Karin S. Coyne

**Affiliations:** 1 Evidera Inc., 7101 Wisconsin Ave., Suite 1400, Bethesda, MD 20814 USA; 2 AbbVie Inc., North Chicago, IL USA; 3 Colorado Center for Reproductive Medicine, Lone Tree, CO USA

## Abstract

**Background:**

Endometriosis is a common problem in women of reproductive age and has impacts on health-related quality of life and productivity. Fatigue is an important part of the burden of endometriosis, it is not often included as an endpoint in clinical trials.

**Objectives:**

The study assessed the psychometric properties of the PROMIS Fatigue Short Form 6a in women with moderate-to-severe endometriosis-associated pain.

**Methods:**

In a phase III double-blind, placebo-controlled clinical trial (NCT01620528), women aged 18–49 years with moderate-to-severe endometriosis-related pain were randomized to elagolix 150 mg once daily, elagolix 200 mg twice daily, or placebo for 6 months. PROMIS Fatigue and dysmenorrhea and non-menstrual pelvic pain (NMPP) scores were assessed at baseline and months 1, 3, and 6, and Patient Global Impression of Change (PGIC) was assessed at months 1, 3, and 6. Reliability (internal consistency and test-retest reliability), construct validity (convergent and known groups validity), and responsiveness were evaluated.

**Results:**

The analysis included 871 women, mean age 31.5 years. Internal consistency supported a single concept (Cronbach’s alpha 0.93). For the 238 patients with no change in PGIC at month 1, the intraclass correlation coefficient for the PROMIS Fatigue T-score was 0.7 and paired t-test statistically significant (2.84, *p* = 0.0049). Correlations with other measures were expected to be fairly low as concepts were not redundant. The PROMIS Fatigue discriminated among known groups with mean scores of 55.3, 62.3, and 65.8 at month 3 (PGIC improvement, no change, worsening, respectively). Statically significant discrimination, and change score responsiveness, were seen using clinically relevant anchors (dysmenorrhea and NMPP) at months 3 and 6 between responders and non-responders. Anchor-based (PGIC) responsiveness showed significant improvement from baseline to months 3 and 6 (*p* < 0.0001).

**Conclusions:**

PROMIS Fatigue has good reliability, validity, and responsiveness in women with moderate-to-severe endometriosis-associated pain.

## Background

Endometriosis is a common problem in women of reproductive age [1, 2]. In the Unites States, endometriosis affects approximately 6%–10% of women at some time in their life. Symptoms include intermenstrual bleeding, non-menstrual pelvic pain (NMPP), and pain during menstruation, intercourse, urination, and defecation. In addition to pain and bleeding, fatigue is experienced by 50%–87% of women with endometriosis and is considered by women to be one of the most burdensome symptoms [3–5]. Due to these symptoms, and their association with infertility, endometriosis has far-reaching consequences on a woman’s health-related quality of life, interfering with marital and sexual relationships, social life, employment, physical activities, and psychological function [4–11].

Although fatigue is an important part of the burden of endometriosis, it is not often included as an endpoint in clinical trials. Several options are available to assess patient-reported fatigue, including the Fatigue Severity Scale [12], Fatigue Impact Scale [13], or Brief Fatigue Inventory [14], but these have not been developed specifically for or been assessed for use among women with endometriosis. The Patient-Reported Outcome Measurement Information System (PROMIS), includes fatigue-related item banks and several fatigue short forms that have been developed and assessed for performance in chronic conditions [15].

A recent qualitative study in women with moderate-to-severe endometriosis-associated pain supported the concept of fatigue as important in this population [16]. In addition, PROMIS Fatigue SF-6a was used as a secondary endpoint to measure patient-reported fatigue in EM-I, a phase III randomized, placebo-controlled clinical trial that assessed the safety and efficacy of elagolix in 871 women with moderate-to-severe endometriosis-related pain [17]. At baseline, 54%–74% of the patients reported frequently having fatigue-related issues. The study also showed that at 6 months, PROMIS Fatigue SF-6a T-scores decreased significantly more in women treated with either dose of elagolix than in women who received placebo, and decreased more in women reporting clinically meaningful reductions in dysmenorrhea, NMPP, and dyspareunia than in women who did not [18].

These findings support the possibility of using PROMIS Fatigue SF-6a as an appropriate endpoint in clinical trials of endometriosis treatments. Here, we describe the psychometric characteristics of PROMIS Fatigue SF-6a in this sample of women with endometriosis based on data collected during the EM-I trial.

## Methods

### Data source and sample

This analysis was based on data collected during the EM-I phase III clinical trial (NCT01620528) [17]. Participants in the trial were premenopausal women from the United States and Canada aged 18–49 years with a surgical diagnosis of endometriosis in the previous 10 years and enrolled from July 2012 to May 2014. Women were excluded if they had clinically significant gynecological conditions other than endometriosis; or chronic pain unrelated to endometriosis [17].

The study ran from May 22, 2012 to September 28, 2015. Participants were randomized 3:2:2 to placebo, elagolix 150 mg once daily, or elagolix 200 mg twice daily. The study included a washout period for women receiving hormonal therapies, a screening period of up to 100 days, and a 6-month treatment period. The co-primary endpoints were the proportions of women with a clinical response for dysmenorrhea and a clinical response for NMPP at 3 months. Secondary endpoints assessed in the current analysis included PROMIS Fatigue SF-6a, Endometriosis Health Profile-30 (EHP-30), the Health Related Productivity Questionnaire (HRPQ), severity of dysmenorrhea and NMPP, and the Patient Global Impression of Change (PGIC) at months 1, 3, and 6. Data on daily analgesic medication use was collected using an electronic diary.

### PROMIS Fatigue SF-6a

PROMIS Fatigue SF-6a is a 6-item instrument with a recall period of last 7 days [19]. Items include (1) “I feel fatigued”; (2) “I have trouble starting things because I am tired”; (3) “How run-down did you feel on average”; (4) “How fatigued were you on average?”; (5) “How much were you bothered by your fatigue on average?”; (6) “To what degree did your fatigue interfere with your physical functioning”. Response options for each item range from “Not at all” (1 point) to “Very much” (5 points). The raw overall (range 6–30) and can be converted to a T-score; higher scores indicate greater fatigue [20]. A T-score more than one standard deviation (10 points) higher than the standardized mean of 50 for the United States population indicates worse than average fatigue than the United States norm.

### EHP-30

EHP-30 is a 30-item disease-specific health-related quality of life instrument comprising five core domains (pain, control and powerlessness, emotional well-being, social support, and self-image) with a recall period of 4 weeks [21]. In addition to the core domains, the clinical study included the optional 5-item sexual relationship module of the EHP-30. Women were able to report if the whole sexual relationship module, or individual items, were not relevant. The EHP-30 was scored according to the developer’s manual where item responses map 0 = Never to 4 = Always and domain scores are standardized to a 0 (best health status) to 100 (worst health status) range. Domain scores were calculated from the sum of the raw scores per domain divided by the maximum possible raw score of items in the domain, multiplied by 100 [22]. The measure was used to characterize the sample’s health-related quality of life impact and the pain domain used to assess construct validity and used as a responsiveness anchor.

### HRPQ

HRPQ is a 9-item questionnaire to assess loss of productivity due to absenteeism and presenteeism [23] assessed for use in women with endometriosis [24]. The questionnaire uses skip patterns to ensure that items are applicable to those who work outside the home (e.g. in full- or part-time employment) and those who work at home.

### Dysmenorrhea and NMPP

During the trial, participants completed a daily electronic diary. Dysmenorrhea was assessed with the item “Choose the item that best describes your pain during the last 24 hours you had your period” and NMPP with the item “Choose the item that best describes your pain during the last 24 hours without your period”. Possible responses included “None” (No discomfort), “Mild” (Mild discomfort but I was easily able to do the things I usually do.), “Moderate” (Moderate discomfort or pain. I had some difficulty doing the things I usually do.), and “Severe” (Severe pain. I had great difficulty doing the things I usually do.). Responses were assigned scores as follows: 0, none; 1, mild, 2; moderate; 3, severe. Scores were averaged over the 35 calendar days immediately prior to and including the date of the first dose of study drug, as well as over the 28 days before each post-baseline assessment. As reported by Taylor and colleagues [17], a dysmenorrhea response was defined as no increase in analgesic use and a score change from baseline of at least − 0.81 (dysmenorrhea responder; if not reached then dysmenorrhea non-responder) and a NMPP response was defined as no increase in analgesic use and a score change from baseline of at least − 0.36 (NMPP responder; if not reached then NMPP non-responder). The thresholds were identified using receiver operating characteristic analysis using the PGIC as an anchor and evaluating changes in analgesic use.

### PGIC

PGIC was assessed using the question “Since I started taking the study medication, my endometriosis related pain has: very much improved (1), much improved (2), minimally improved (3), no change (4), minimally worse (5), much worse (6), very much worse (7).”

### Statistical analyses: general considerations

All analyses were performed in all randomized subjects who received at least one dose of treatment or placebo. Missing data were not imputed. SAS version 9.4 (SAS Institute, Cary, NC, USA) and Mplus version 7.11 (Los Angeles, CA) were used to perform analyses.

#### Assessment of ceiling and floor effects

Ceiling effects were explored based a highest response option and floor effects based on a lowest response option.

#### Confirmatory factor analysis

A categorical confirmatory factor analysis using polytomous response options was performed to evaluate the fit of the PROMIS Fatigue SF-6a as a unidimensional fatigue scale. Model fit was assessed using recommendations by Reeve and colleagues [25]. Specifically, the model fit was evaluated by considering the comparative fit index (suggested cut point > 0.95); Tucker-Lewis Index (suggested cut point > 0.95); weighted root mean square residual (suggested cut point < 1.0); average absolute residual correlations (suggested cut point < 0.10); root mean square error of approximation (suggested cut point < 0.06).

#### Assessment of reliability

Internal consistency reliability of the PROMIS Fatigue was assessed by calculating Cronbach’s alpha for data collected at baseline. Values above 0.70 are generally considered acceptable for aggregate data [26]. Item performance for the PROMIS Fatigue SF-6a was evaluated by calculating Cronbach’s alpha with individual items deleted. Test-retest reliability (reproducibility) of PROMIS Fatigue SF-6a was assessed in patients with no change in PGIC at month 1; T-scores were compared between baseline and month 1 by paired t-test and intra-class correlations.

#### Assessment of construct validity

Convergent validity was assessed by calculating Pearson product-moment and Spearman’s rank correlation coefficients at baseline, month 3, and month 6 for PROMIS Fatigue SF-6a versus dysmenorrhea, NMPP, HRPQ, and EHP-30. Because the concepts being measured are not redundant, rather hypothesized to be distally related, the expectation is that positive, low to moderate correlations are expected. Cohen’s conventions were used when interpreting correlation coefficients as 0.10 small, 0.30 moderate, and 0.50 large [27].

Known groups validity was assessed by comparing PROMIS Fatigue SF-6a T-score at months 3 and 6 between the following subgroups: dysmenorrhea and NMPP responders versus non-responders; and PGIC (improved; no change; worsened). Responder status (responder or non-responder) for dysmenorrhea and NMPP was from the Endometriosis Daily Pain Impact diary score as noted in the clinical study [17]. General linear models (Proc GLM) were used to calculate F statistics and *p*-values; for multiple group pairwise comparisons Scheffe’s test was used to adjust for the multiple comparisons.

#### Assessment of responsiveness

The PROMIS Fatigue SF-6a was explored using a triangulation approach comprising anchor-based analyses, difference between means analyses, and use of clinically relevant indicators to test the instrument’s ability to detect change during the clinical study. Responsiveness is the ability of a measure to detect change when change is present [28].

In anchor-based analyses, the least-squares (LS)-mean change from baseline in PROMIS Fatigue SF-6a T-score at months 3 and 6 were compared for the following subgroups: PGIC (improved, no change, worsened) and EHP-30 pain domain responders (≥30-point decrease in EHP-30 pain domain score from baseline) versus non-responders. General linear models controlling for age and baseline PROMIS Fatigue score were used to calculate F statistics and *p*-values using Scheffe’s test to adjustment for multiple comparisons.

In the assessment of standardized difference between two means, effect size (Cohen’s d), was calculated for PROMIS Fatigue SF-6a at months 3 and 6. The analyses do not include direct patient feedback, thus cannot serve as the primary assessment for within-patient clinical meaningfulness, and should be considered only as supportive [29]. Effect size was calculated by subtracting the baseline score from the post-baseline (month 3 or 6) score and dividing the result by the baseline standard deviation. As described by Cohen [27], effect sizes were classified as small (0.20), moderate (0.50), or large (0.80). Change from baseline was analyzed by paired t-test.

Clinically relevant indicators were used to explore the responder threshold, which was defined as the LS-mean change in PROMIS Fatigue SF-6a score from baseline that indicated a meaningful response to treatment. Responder thresholds for PROMIS Fatigue SF-6a T-score at 3 and 6 months were calculated for dysmenorrhea and NMPP responders and non-responders.

## Results

### Sample characteristics

This analysis included the 871 participants enrolled in the EM-I trial who received at least one dose of study treatment or placebo (Table 1). Mean age was 31.5 years and most were White (87.1%) and not Latino (84.0%). The majority of the women were employed (60.2% full time; 17.0% part-time). For the EHP-30 domains, mean scores at baseline were 58.2 (14.3) for pain, 49.3 (19.3) for emotional well-being, 69.8 (19.5) for control and powerlessness, 54.8 (25.6) for social support, 51.0 (27.6) for self-image, and 64.5 (24.7) for sexual relationships. The mean PROMIS Fatigue T-score at baseline was 63.3 (7.7) (range, 33.4–76.8).

**Table 1:** Sociodemographic characteristics and patient-reported data at baseline

Characteristic	Total (***N*** = 871)
**Age (Mean, SD)**	31.5 (6.2)
**Race (n, %)**	
White	759 (87.1%)
Black or African American	76 (8.7%)
Asian	9 (1.0%)
American Indian or Alaska Native	6 (0.7%)
Multi Race	18 (2.1%)
Native Hawaiian or Other Pacific Islander	3 (0.3%)
**Ethnicity (n, %)**	
Hispanic or Latino	139 (16.0%)
Not Hispanic or Latino	732 (84.0%)
**Employment Status**^**a**^	
Employed full-time	524 (60.2%)
Employed part-time	148 (17.0%)
Non-Employed	193 (22.2%)
Missing	6 (0.7%)
**EHP-30 Domain Scores**^**b**^	
Pain	58.2 (14.3) [0.0–100.0]
Control and Powerlessness	69.8 (19.5) [0.0–100.0]
Emotional Well-being	49.3 (19.9) [0.0–100.0]
Social Support	54.8 (25.6) [0.0–100.0]
Self-image	51.0 (27.6) [0.0–100.0]
Sexual Relationship	64.5 (24.7) [0.0–100.0]
**PROMIS Fatigue Short Form 6a**^**c**^	63.3 (7.7) [33.4–76.8]

*EHP-30* Indicates Endometriosis Health Profile-30, *PROMIS* Patient-Reported Outcome Measurement Information System, *SD* Standard deviation

^a^Reported in the HRPQ first item

^b^Each domain has a 0–100 scale range where 0 indicates the best health status

^c^T-score Higher scores indicating more fatigue; population mean is 50 and 10 is one standard deviation from the general population

### Ceiling and floor effects

A ceiling effect was observed for one PROMIS Fatigue SF-6a item at baseline, “How much were you bothered by your fatigue on average?”, with 32.6% responding “Very much”. No other ceiling or floor effects were detected at baseline or at months 3 or 6.

### Confirmatory factor analysis

Confirmatory factor analysis demonstrated strong fit for a unidimensional scale with item factor loadings ranging between 0.808–0.942 (Table 2). Model fit was supported by the comparative fit index, Tucker-Lewis index, and all absolute residual correlations less than 0.10. The data were nonnormative and the weighted root mean square residual and RMSEA estimate was 0.112 (90% confidence interval, 0.093–0.132) which is higher than the acceptable value but not uncommon for small degrees of freedom [30, 31].

**Table 2:** Confirmatory factor analysis PROMIS Fatigue 6a at baseline

**PROMIS Fatigue Items**	**Factor Loadings**
1. I feel fatigued	0.863
2. I have trouble starting things because I am tired	0.808
3. How run-down did you feel on average?	0.879
4. How fatigued were you on average?	0.942
5. How much were you bothered by your fatigue on average?	0.867
6. To what degree did your fatigue interfere with your physical functioning?	0.842
**Model Fit**	**Statistic**
Comparative Fit Index	0.994
Tucker-Lewis Index	0.991
Weighted Root Mean Square Residual	1.074
Absolute Residual Correlations	all < 0.10
RMSEA Estimate	0.112
RMSEA Lower 90% Confidence Limit	0.093
RMSEA Upper 90% Confidence Limit	0.132

*PROMIS* Indicates Patient-Reported Outcome Measurement Information System, *RMSEA* Root mean square error of approximation

### Reliability

Cronbach’s alpha was 0.93 at baseline and 0.91–0.92 when individual items were deleted, indicating that the items comprising PROMIS Fatigue SF-6a measured the same construct. In the 238 patients with no change in PGIC at month 1, the interclass correlation for baseline versus month 1 was 0.7 and paired t-test were statistically significant (t-value 2.84, *p* = 0.0049) for the PROMIS Fatigue T-score indicating stable test-retest reliability.

### Construct validity

Spearman’s rank correlation coefficients indicated a moderate correlation at baseline between PROMIS Fatigue SF-6a and the EHP-30 pain domain (0.34) and weak correlations between PROMIS Fatigue SF-6a and HRPQ work absenteeism (0.22), HRPQ work presenteeism (0.23), dysmenorrhea (0.17), and NMPP (0.17) (Table 3). At month 3, Spearman’s rank correlation coefficients ranged from 0.27 (dysmenorrhea) to 0.49 (EHP-30 pain domain), and at month 6, they ranged from 0.35 (HRPQ work absenteeism) to 0.60 (EHP-30 pain domain). Pearson product-moment correlations were found to be similar to the Spearman’s rank correlation coefficients (Table 3).

**Table 3:** Correlations (Spearman and Pearson) between PROMIS Fatigue Short Form 6a and patient-reported outcome measures at baseline, month 3, and month 6

Measure	Baseline		Month 3		Month 6	
	N	PROMIS Fatigue (Spearman, Pearson)	N	PROMIS Fatigue (Spearman, Pearson)	N	PROMIS Fatigue (Spearman, Pearson)
EHP-30 pain domain	851	(0.34, 0.34)	719	(0.49, 0.51)	576	(0.60, 0.60)
Dysmenorrhea over the last 28 days	860	(0.17, 0.17)	732	(0.27, 0.28)	573	(0.39, 0.38)
NMPP over the last 28 days	860	(0.17, 0.17)	732	(0.36, 0.37)	573	(0.49, 0.46)
HRPQ hours of absenteeism from work due to endometriosis	656	(0.22, 0.17)	498	(0.33, 0.30)	373	(0.35, 0.30)
HRPQ hours of presenteeism from work due to endometriosis	623	(0.23, 0.20)	491	(0.41, 0.36)	368	(0.45, 0.37)

*EHP-30* Indicates Endometriosis Health Profile-30, *HRPQ* Health Related Productivity Questionnaire, *NMPP* Non-menstrual pelvic pain, *PROMIS* Indicates Patient-Reported Outcome Measurement Information System, *SD* Standard deviation

### Known groups validity

The mean PROMIS Fatigue T-scores at month 3 were significantly lower for dysmenorrhea and NMPP responders than for non-responders (Table 4). At month 3 the dysmenorrhea responders mean score was 54.5 and the non-responders was 59.1; the DYS responder mean was 54.4 and the non-responders was 59.8. Similar results for mean PROMIS Fatigue T-score between clinical responders and non-responders were seen at month 6. The mean PROMIS Fatigue T-score was also significantly lower in participants for whom PGIC showed improvement than in those for whom PGIC did not change (55.3 vs. 62.3, *p* < 0.001) or worsened (55.3 vs. 65.8, *p* < 0.001) at month 3; results at month 6 were similar (Table 5).

**Table 4:** Known groups validity: PROMIS Fatigue Short Form 6a T-score by dysmenorrhea and NMPP responder status at months 3 and 6

Time	Outcome	Responders		Non-responders		F-test^a^	
		N	Mean (SD)	N	Mean (SD)	F	***P***-value
Month 3	Dysmenorrhea^b^	351	54.5 (10.0)	381	59.1 (8.8)	42.78	< 0.0001
	NMPP^c^	397	54.4 (9.5)	335	59.8 (9.0)	62.62	< 0.0001
Month 6	Dysmenorrhea^b^	292	52.9 (10.2)	281	59.7 (8.8)	73.64	< 0.0001
	NMPP^c^	321	53.2 (10.0)	252	60.2 (8.8)	78.10	< 0.0001

*NMPP* Non-menstrual pelvic pain, *PROMIS* Patient-Reported Outcome Measurement Information System, *SD* Standard deviation

^a^General linear model controlling for age and baseline Fatigue score

^b^Pain due to endometriosis over the last 28 days during menstruation

^c^Pain due to endometriosis over the last 28 days when the patient was not menstruating

**Table 5:** PROMIS Fatigue Short Form 6a by PGIC: improved, no change, and worsened

Time	Value	PGIC improved^a^		No change in PGIC		PGIC worsened^b^		Overall F-test^c^		***P***-value for pairwise comparisons^d^	
		N	Mean (SD)	N	Mean (SD)	N	Mean (SD)	F	***P***-value	Improved vs. no change	Improved vs. worsened
Month 3	Score	571	55.3 (9.2)	111	62.3 (7.8)	44	65.8 (10.6)	49.8	< 0.0001	< 0.001	< 0.001
	Change from baseline	565	−7.9 (0.4)	109	−0.9 (0.8)	44	2.3 (1.3)	69.20	< 0.0001	< 0.001	< 0.001
Month 6	Score	404	54.3 (9.9)	81	60.6 (7.6)	58	63.9 (9.2)	35.2	< 0.0001	< 0.001	< 0.001
	Change from baseline	398	−8.9 (0.4)	79	−3.1 (1.0)	58	0.6 (1.2)	48.54	<.0001	< 0.001	< 0.001

*PGIC* Indicates Patient Global Impression of Change, *PROMIS* Patient-Reported Outcome Measurement Information System, *SD* Standard deviation

^a^Very much improved, much improved, or minimally improved

^b^Minimally worse, much worse, or very much worse

^c^General linear model, controlling for age and baseline PROMIS Fatigue SF 6a score for change values

^d^Scheffe’s test adjusting for multiple comparisons

### Responsiveness

Two anchor-based approaches were used to evaluate PROMIS Fatigue SF-6a responsiveness, the patient-reported changes in PGIC (improved, no change, worsened) and responder status on the EHP-30 pain domain. At month 3 the mean changes in the PROMIS Fatigue SF-6a T-score were − 7.9 (0.4) for participants who reported an improvement, − 0.9 (0.8) for participants who reported no change, and 2.3 (1.3) for participants who reported a worsening using the PGIC (Table 5). The findings demonstrate a PROMIS Fatigue SF-6a score response when a change is identified. Similar findings were seen at month 6 using the PGIC as an anchor. At month 3 and 6 the PROMIS Fatigue SF-6a T-scores were significantly different for those who were a responder versus not a responder on the EHP-30 pain domain (*p* < 0.0001 at both timepoints). The mean T-score difference for the EHP-30 responders versus non-responders was − 10.3 (0.5) and − 3.5 (0.4) at month 3 and − 11.8 (0.5) and − 3.3 (0.5) at month 6.

The responsiveness in distribution-based analyses uses only the PROMIS Fatigue SF-6a data to look for changes over time and does not include outside sources of data such as the PGIC or clinical indicator. The treatment groups should have a change, an improvement, between baseline and month 3 and month 6. It is also expected that the placebo group may have an improvement in PROMIS Fatigue SF-6a scores over time due to placebo effect. Table 6 reports the responsiveness findings for the total sample then by treatment arms. For all groupings, at both timepoints, there is a reduction in fatigue indicating less fatigue. The total sample of participants had a − 6.2 (9.8) change from baseline to month 3 in their mean PROMIS Fatigue SF-6a T-score the change was a significant decrease (*p* < 0.0001); at month 6 the total sample score changed − 6.8 (10.4) and was also a significant decrease (*p* < 0.0001).

**Table 6:** Responsiveness of PROMIS Fatigue Short Form 6a at months 3 and 6

Time	Sample	N	Mean (SD)			t-value (***p***-value)^**†**^	Effect size (Cohen’s d)^b^
			Baseline	Post-baseline^a^	Change (SD)		
Month 3	Total Sample	728	63.2 (7.5)	57.0 (9.7)	−6.2 (9.8)	17.12 (< 0.0001)	−0.83
	Placebo	314	62.4 (7.4)	58.2 (9.7)	−4.2 (9.3)	8.02 (<.0001)	−0.57
	150 mg	216	64.0 (7.8)	57.4 (9.5)	−6.7 (10.0)	9.79 (<.0001)	−0.85
	200 mg	198	63.6 (7.3)	54.6 (9.5)	−9.0 (9.9)	12.90 (<.0001)	−1.25
Month 6	Total Sample	577	63.1 (7.5)	56.3 (10.1)	−6.8 (10.4)	15.76 (< 0.0001)	−0.91
	Placebo	246	62.5 (7.7)	58.3 (10.0)	−4.2 (9.8)	6.73 (<.0001)	−0.55
	150 mg	170	63.6 (7.7)	56.6 (9.4)	−7.0 (9.9)	9.24 (<.0001)	−0.91
	200 mg	161	63.5 (7.2)	52.9 (10.0)	−10.6 (10.7)	12.64 (<.0001)	−1.48

*PROMIS* Indicates Patient-Reported Outcome Measurement Information System, *SD* Half standard deviation, *SEM* Standard error of the mean

^†^Paired t-test

^a^Month 3 or month 6

^b^Calculated as mean change / SD for baseline score

**Fig. 1:**
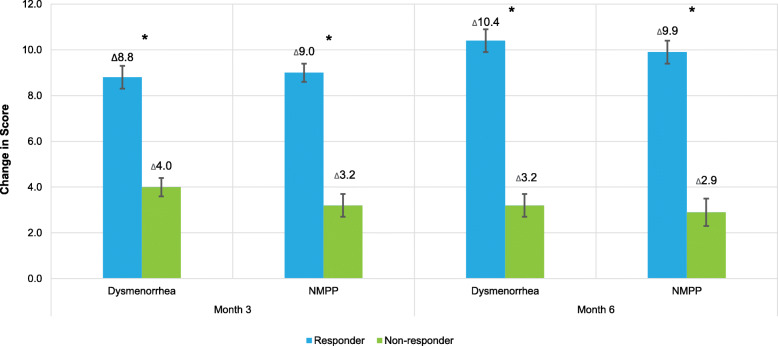
Clinically Relevant Responsiveness for PROMIS Fatigue Short Form 6a Change for Dysmenorrhea and NMPP Responders at Month 3 and Month 6. LS-mean indicates least-squares mean; NMPP, non-menstrual pelvic pain; PROMIS, Patient-Reported Outcome Measurement Information System; SE, standard error. * *P* value between responder and non-responder is *p* < 0.0001

Clinically relevant indicators were used to assess the responsiveness of the PROMIS Fatigue SF-6a. For dysmenorrhea responders, the LS-mean change from baseline in PROMIS Fatigue SF-6a T-score was significantly greater than those who did not have a response to treatment in dysmenorrhea (Fig. 1). Dysmenorrhea responders had a score change of − 8.8 (0.5) versus dysmenorrhea non-responders having a change of − 4.0 (0.4) at month 3 and − 10.4 (0.5) versus dysmenorrhea non-responders change of − 3.2 (0.5) at month 6 (both comparisons *p* < 0.0001). NMPP responders versus non-responders had significant score changes for the responders versus the non-responders at both timepoints too (Fig. 1).

## Discussion

This analysis showed that for women with moderate-to-severe endometriosis-associated pain, PROMIS Fatigue SF-6a performs well, with evidence of reliability, construct validity, and responsiveness to change. The instrument measures a single construct, and the resulting scores could discriminate between pain severity groups, between responders and non-responders for clinically relevant endpoints (dysmenorrhea and NMPP), and between levels of patient self-assessed global change. Correlations with other patient-reported outcomes were moderate to weak at baseline, indicating that PROMIS Fatigue SF-6a is not redundant with other patient-reported outcomes.

For a patient-reported outcome measure to be fit for purpose there needs to be evidence that the concept being measured is appropriate and applicable to the target population and that the measure performs well in the target population. The current findings compliment the results of a previous study demonstrating content validity and appropriateness of PROMIS Fatigue SF-6a in this population [16]. They also provide quantitative evidence about the positive performance (reliability, validity, and responsiveness) of the instrument, therefore supporting a recent study based on PROMIS Fatigue SF-6a data from the EM-I trial, which showed that the women in the trial frequently experienced severe fatigue at baseline and that elagolix significantly improved fatigue after 6 months of treatment [18].

The mean T-score at baseline in this study was more than one standard deviation higher than the standardized mean for the United States population, indicating that the women included in this analysis experienced significantly greater fatigue than the US norm at study baseline. To put this in context, the baseline T-scores in this study were higher than reported for back pain, cancer, chronic heart failure, chronic obstructive pulmonary disease exacerbation, stable chronic obstructive pulmonary disease, major depressive disorder, and rheumatoid arthritis, muscular dystrophy, multiple sclerosis, post-polio syndrome, spinal cord injury, and chronic pelvic pain [32–34]. The baseline T-score in the present analysis was also close to the T-score obtained using the PROMIS Fatigue Short Form 7a in a recent study of adults with myalgic encephalomyelitis and chronic fatigue [35].

PROMIS Fatigue Short Form 7a has been similarly shown to have sound psychometric properties in pregnant women and patients with fibromyalgia, sickle cell disease, and cardio-metabolic risk [36].

The patient-focused drug development guidance series from the US Food and Drug Administration has provided insight to the Agency’s views about the development and use of patient-reported outcome measures as clinical study endpoints. The first draft guidance focuses on the comprehensive and representative input during product development [37]. The guidance details the patient experience data includes information about the *experience* and *impact* a condition has on the patient. The concept of fatigue has been shown to be important to this target population and the use of a measure that assesses the concept is appropriate. Having the ability to reliably measure fatigue among women with moderate-to-severe endometriosis-related pain can be an important from a clinical and humanistic perspective. The PROMIS Fatigue SF-6a can measure changes in fatigue and add value in clinical practice and research. This research is an example of how an existing measure can be assessed for use in a new population. The assessment of the psychometric properties and responsiveness support the use of the PROMIS Fatigue SF-6a among women with moderate-to-severe endometriosis-related pain. The findings from this research could be used to identify a responder threshold that would indicate a treatment benefit among this target sample. Previous research with different versions of the Short Form of the PROMIS (e.g. 17 item Short form) has suggested a change of 3–5 points to indicate a responder [38].

A strength of this study is that the data were from a large randomized, controlled trial and were therefore of high quality. At the same time, the results may not be generalizable outside of the selected population, which may have a different racial makeup than the overall population of women in the US with endometriosis, or to women with more mild endometriosis-associated pain. In addition, the results may not be generalizable to other PROMIS Fatigue measures for this population or to the use of PROMS Fatigue SF-6a in other women’s health conditions.

## Conclusion

In conclusion, this study showed that PROMIS Fatigue SF-6a performs well in women with moderate-to-severe endometriosis-related pain, with good reliability, validity, and responsiveness to change. The study also confirmed that fatigue is a common and severe problem in women with endometriosis, highlighting the need for a high-quality instrument for assessing fatigue as a treatment outcome in this population.

## Data Availability

AbbVie is committed to responsible data sharing regarding the clinical trials we sponsor. This includes access to anonymized, individual and trial-level data (analysis data sets), as well as other information (e.g., protocols and Clinical Study Reports), as long as the trials are not part of an ongoing or planned regulatory submission. This includes requests for clinical trial data for unlicensed products and indications. This clinical trial data can be requested by any qualified researchers who engage in rigorous, independent scientific research, and will be provided following review and approval of a research proposal and Statistical Analysis Plan (SAP) and execution of a Data Sharing Agreement (DSA). Data requests can be submitted at any time and the data will be accessible for 12 months, with possible extensions considered. For more information on the process, or to submit a request, visit the following link: https://www.abbvie.com/our-science/clinical-trials/clinical-trials-data-and-information-sharing/data-and-information-sharing-with-qualified-researchers.html

## Abbreviations

EHP-30: Endometriosis Health Profile-30
HRPQ: Health Related Productivity Questionnaire
LS: Least-squares
NMPP: Non-menstrual pelvic pain
PGIC: Patient Global Impression of Change
Proc GLM: General linear models
PROMIS: Patient-Reported Outcome Measurement Information System
RMSEA: Root mean square error of approximation
SD: Standard deviation
SEM: Standard error of the mean
SF-6a: ROMIS Fatigue Short Form 6a
